# Characterization and Optimization of Real-Time Photoresponsive Gelatin for Direct Laser Writing

**DOI:** 10.3390/polym14122350

**Published:** 2022-06-09

**Authors:** Branka D. Murić, Dejan V. Pantelić, Mihajlo D. Radmilović, Svetlana N. Savić-Šević, Vesna O. Vasović

**Affiliations:** 1 Institute of Physics, University of Belgrade, Pregrevica 118, 11080 Belgrade, Serbia; pantelic@ipb.ac.rs (D.V.P.); mihajlor@ipb.ac.rs (M.D.R.); savic@ipb.ac.rs (S.N.S.-Š.); 2 Western Serbia Academy of Applied Studies, Užice Department, Trg Svetog Save 34, 31000 Užice, Serbia; bolex65@yahoo.com

**Keywords:** gelatin, bio-eco-polymers, physicochemical properties, optical elastomers, rapid laser printing, microstructures

## Abstract

There is an abundance of plastic materials used in the widest range of applications, such as packaging, machine parts, biomedical devices and components, etc. However, most materials used today are non-decomposable in the environment, producing a huge burden on ecosystems. The search for better, safer alternatives is still on. Here we present a detailed analysis of a simple, cheap, non-toxic, even edible, eco-friendly material, which can be easily manufactured, laser patterned and used for the fabrication of complex structures. The base substance is gelatin which is made photoresponsive by adding plasticizers and sensitizers. The resulting films were analyzed with respect to their optical, thermal and mechanical properties, which can be modified by a slight variation of chemical composition. The material is optimized for rapid laser-manufacturing of elastic microstructures (lenses, gratings, cantilevers, etc.) without any waste or residues. Overall, the material properties were tailored to increase photothermal responsivity, improve the surface quality and achieve material homogeneity, transparency and long-term stability (as verified using electron microscopy, infrared spectroscopy and differential scanning calorimetry).

## 1. Introduction

The mechanical strength, low production cost and manufacturability of petroleum-based plastics established them as the most popular packaging materials [1]. Unfortunately, their non-biodegradability makes them an important source of pollution [2]. The ecological impact of petroleum-based plastics has accelerated the worldwide interest in biodegradable polymers from renewable resources. Despite the great potential of biodegradable polymers to solve the problem of plastic waste, they are rarely used, considering that their production is relatively expensive and they do not possess all the other properties (e.g., mechanical, optical, and electrical) required by various end-applications [3]. Additionally, such materials may have quite variable properties. For example, tensile strength, strain at break and elasticity (Young’s modulus) depend on the raw material source, additives, molecular structures, etc. [4,5].

A good example of a natural biodegradable polymer from renewable resources is starch which is inexpensive, readily available and biodegradable. However, its mechanical strength is strongly modulated by its moisture sensitivity while being difficult for thermal processing (it thermally decomposes before being melted). To modify these characteristics, starch is blended with other polymers [6,7,8]. Unfortunately, these blends can affect biodegradability. Furthermore, there is a direct relationship between the starch-based bioplastic properties and different starch botanical sources. Namely, the amylose and amylopectin proportion, which depends on starch biosynthesis enzymes, soil type, and climatic conditions during plant growth, strongly influences the bioplastic gelation temperature and mechanical and rheological properties [9]. Nevertheless, significant research and technological work invested in the development of starch-based polymers in the packaging sectors of food, cosmetics, pharmaceuticals, etc. [10,11,12].

Gelatin, yet another biocompatible and biodegradable polymer, is widely used in photography, food, pharmaceutical, biomedical and many others applications [13,14,15,16,17]. It has poor mechanical properties that limit its potential applications. A variety of chemical agents (formaldehyde, epoxy, glutaraldehyde and some others) are capable of crosslinking gelatin, consequently overcoming the brittleness of gelatin films, improving its flexibility, reducing the degree of swelling and enhancing its thermal stability. Glutaraldehyde [18] is usually used but is being replaced with genipin because of its lower cytotoxicity [19,20].

Besides gelatin, many other natural polymers (such as albumen, alginate, cellulose, chitosan, pullulan, etc.) can be used too [21,22]. We emphasize that the photoresponsiveness and photosensitivity of most materials stem from harmful and poisonous chemicals (such as dichromate compounds).

Also, gelatin is often mixed with other substances, e.g., chitosan, poly(vinyl alcohol), alginate and carbon nanotubes. These hybrid materials are specifically of interest in biomedicine for drug delivery, wound healing, cell culture and tissue engineering [23,24,25].

Gelatin hydrogels are photosensitized by various dyes, making them suitable for laser writing of microstructures (such as microfluidic channels, microlenses, micro-optoelectromechanical systems (MOEMS), lab on a chip, etc.) intended for a wide variety of applications [26,27,28]. Gelatin was widely used previously in optics, primarily as a photosensitive material for holography. Photo-crosslinking was achieved by adding ammonium dichromate or potassium dichromate, which resulted in holograms of excellent quality. It is known that gelatin doped with ammonium dichromate (dichromated gelatin—DCG) represents an almost ideal high-resolution holographic photosensitive material [29]. Additionally, DCG can be used as a material for the production of micro-optical components [30,31]. Most of them are toxic, difficult to manufacture and require complex chemical postprocessing. Various silicate-based materials (silicones, silica glass, etc.) were used for the fabrication of micro-optical components [32,33]. Compared to gelatin, they have higher thermal and chemical stability, but the fabrication processes are manly complex, multistep, time-consuming and costly, frequently including the use of poisonous chemicals.

Emerging applications in biomedicine, micro-mechanics, micro-optics, energy storage, and sensors require new materials, simultaneously possessing advanced optical, electrochemical, mechanical and biological properties [34,35,36]. Previously, we have described photosensitive tot’hema-gelatin as a good candidate material for applications requiring real-time manufacturing of micro-optical and micro-mechanical components [37,38,39,40]. Gelatin was modified with a complex aqueous mixture of various salts, plasticizers, humectants and preservatives (commercially sold under the trade name tot’hema, which is used to treat iron anemia). The material is simple to prepare, low-cost, non-toxic, biodegradable and can be patterned by direct laser writing (DLW—widely used non-contact high-precision processing technique [41,42]). Here, a variety of micro-optical and microfluidic structures were fabricated using DLW, and threshold laser energy was established. This kind of microprocessing is neither additive nor subtractive as the material is only redistributed without any waste.

Therefore, in order to fully explore and optimize the capabilities of tot’hema-gelatin films for real industrial applications, the physicochemical characteristics of the film were studied in more detail and disclosed in this paper. We correlated the applied concentration of tot’hema with the film properties, such as color, thickness, light transmission, mechanical (tensile strength, elongation at break and Young’s modulus), thermal properties, moisture content, swelling, the functional group on the film surface as well as surface morphology. These properties are essential for establishing optimal conditions of material used as an elastomer for adaptive, rapid prototyping of micro-optics and micro-mechanics.

## 2. Materials and Methods

### 2.1. Materials

All chemical components were analytical grade, well known, easily available, cheap and non-toxic. Commercial gelatin from bovine skin (gel strength ~225 g Bloom, Type B) and NaCl (puriss, p.a.) were purchased from Sigma Aldrich. Tot’hema, an oral solution for the treatment of human anemia, was manufactured by Laboratoire Innotech International, France. According to the manufacturer’s data [43], this is a complex water solution of gluconate (iron, manganese and copper) and excipients (glycerol, glucose liquid, saccharine, citric acid, polysorbate 80, etc.). Saline solution was purchased from Hemofarm (Serbia).

### 2.2. Film Preparation

A 5% water solution of gelatin with added NaCl (20% by weight of dry gelatin) was prepared as described in previous papers [37,38]. The tot’hema (ranging from 0% to 30% *v*/*v*) was mixed with gelatin solution by a magnetic stirrer. The series of five solutions (denoted as TG_X, where the letter X indicates the tot’hema percentage in solution) were prepared and centrifuged in order to remove all remaining impurities. 

TG_X films were prepared by the gravity settling method [39]. Namely, the accurately measured volume of TG_X solution was pipetted onto a very clean, horizontally leveled microscope glass slide and dried in a stable environmental condition over the night.

### 2.3. Mechanical Properties

A film thickness was determined using a digital micrometer (an accuracy of 0.01 mm) at eight randomly selected points in the center and edges of the films. Measurements were averaged and taken as the film thickness. 

The surface morphologies of the tot’hema-gelatin films were analyzed using a high-resolution scanning electron microscope equipped with a high brightness Schottky field emission gun (FEGSEM) and using non–contact profilometry (3D optical surface profiler—Zygo New View 7100).

**Figure 1:**
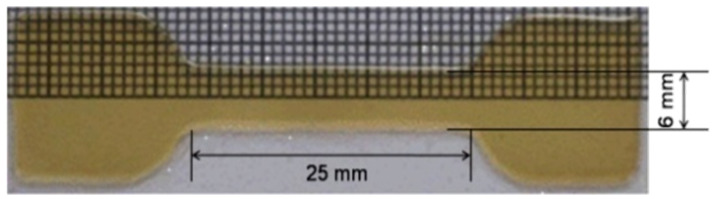
Optically transparent TG_20 film is used for tensile testing with dimensions according to the ASTM standards (gauge length 25 mm, width 6 mm).

The mechanical properties of the films were determined using a tensile testing machine, at a strain rate of 20 mm/min, as previously described for photosensitive tot’hema-gelatin, with minor modifications [44]. The dried film was cut by a brass mold, designed according to the ASTM standard [45], into a dog-bone-shaped specimen (see Figure 1). Three measurements were made for each film, and the average value was calculated. The tensile strength, elongation at break and Young’s modulus were determined.

### 2.4. Optical Properties

The transmittance of the TG_X films was analyzed using a fiber-type spectrometer (Ocean Optics) with a tungsten-halogen light as an illumination source in the wavelength range from 200 nm to 800 nm at room temperature.

A home-made direct laser writing system was used to fabricate microfluidic, micro-mechanic and micro-optical components on tot’hema modified gelatin film. A laser beam (488 nm wavelength, up to 100 mW power–iBEAM SMART, manufactured by Toptica) was focused through the long-working-distance microscope objective. The samples were mounted on an xyz-coordinate stage to enable precise movement of the material along with the arbitrary pattern. The system was computer-controlled using G-code (standard programming language for CNC machines). More details about the system are given in a previous publication [46].

### 2.5. Physicochemical Properties

To determine how the number of water changes during the drying–dehydration of the film, the water content (WC) was measured. The TG_X films were weighted (w_1_), then dried for 24 h at normal laboratory conditions, and weighted again (w_2_). The WC for each film was determined as an average value of three measurements by the equation: $\mathrm{WC}\left(\%\right)=\left[\left(w_{1}-w_{2}\right)/w_{1}\right]\cdot 100$ [47].

In order to determine the influence of tot’hema concentration on the film swelling, TG_X films were immersed in saline solution for different intervals at laboratory conditions. Wet, swollen samples, after wiping with filter paper to remove excess liquid, were weighted again (w_w_). The swelling factor (SF) of the films was calculated as the average value of three measurements by the equation: $\mathrm{SF}\left(\%\right)=\left[\left(w_{w}-w_{d}\right)/w_{d}\right]\cdot 100$ where (w_d_) corresponds to the weight of TG_X films before immersion [48].

Functional groups on the films were inspected by Fourier transformed infrared (FTIR) spectroscopy. The spectra were recorded on a Nicolet iS10 FTIR Spectrometer (Thermo Scientific Instruments) equipped with an attenuated total reflectance (ATR) accessory. ATR/FTIR measurements were done in the wavenumber interval from 500 to 4000 cm^−1^ with a resolution of 4 cm^−1^.

The thermal stability of the films was analyzed by differential scanning calorimetry (DSC). An amount of about 2.5 mg of each film sample was packed in an aluminum pan (30 µL). The pan was sealed and analyzed using a DSC 131 EVO (SETARAM Instrumentation) differential scanning calorimeter previously calibrated with indium. An empty sealed pan was used as a reference. Both pans were placed in a chamber under the nitrogen flow of 20 mL/min, kept at 20 °C for 5 min and subsequently heated from 20 to 250 °C with a heating rate of 10 °C/min. To construct a baseline, two empty pans were placed in the chamber and measured under the same experimental conditions. The baseline subtraction was performed using the CALISTO Processing software (SETARAM Instrumentation).

## 3. Results

**Figure 2:**
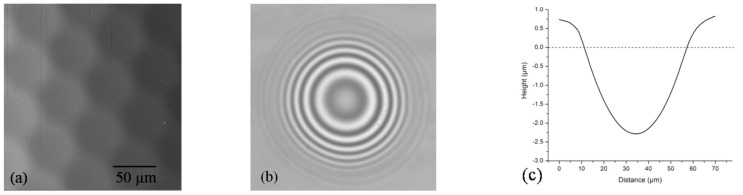
(**a**) SEM image of hexagonal microlenses array; (**b**) Interference fringe pattern of a microlens; and (**c**) 2D surface profile of a microlens.

The pure gelatin film (TG_0) has a uniform, relatively smooth and flat surface, which indicates that the gelatin has a good film-forming ability. We have found that there is no noticeable difference in the gelatin film surface after tot’hema adding. Films still keep a rather smooth, uniform and homogeneous surface (without pores or cracks), indicating that there is good compatibility between tot’hema and gelatin molecules. Smoothness remains high after laser processing, as verified by SEM analysis of hexagonal concave microlenses array fabricated on photoresponsive tot’hema-gelatin film (Figure 2a). The lens-shaped dip is very symmetric (verified by the concentric shape of the corresponding interferogram in Figure 2b), and its roughness is of the order of several tens of nanometers (see Figure 2c). This makes photoresponsive tot’hema-gelatin an excellent material for the fabrication of micro-optical components.

To study mechanical properties, equal volumes of solution were used to prepare all TG_X films. An increase in tot’hema concentration resulted in the film thickening from 25 µm (pure gelatin) to 200 µm (30% of tot’hema) as a result of the plasticizer’s interaction with the polymer chains. Tot’hema added to gelatin increases the distance between protein chains, increasing the film thickness. It can be noticed that the highest tot’hema concentration (30%) results in the thickest film due to the fact that most tot’hema ingredients are hydrophilic molecules that retain water and further increase the film thickness. Moreover, increased tot’hema content in gelatin film decreases the stress at break (reaching a minimum of 0.72 MPa at 30% of tot’hema) and increases the strain at break (turning tot’hema-gelatin into an elastomer—maximum elasticity of about 220% was obtained). Mechanical properties of TG_X films, as a function of the concentration of tot’hema, are summarized in Table 1. The tot’hema addition (which is a mixture of different plasticizers and humectants) to the gelatin matrix improves the film flexibility and, consequently, makes it less rigid. Namely, glycerol is well known as a plasticizer. As a hydrophilic molecule with low molecular weight, glycerol can easily fit into protein chains creating hydrogen bonds [49]. Furthermore, tot’hema provides additional mobility of protein chains by increasing their distance, influencing a significant decrease of Young’s modulus of elasticity, from 1933 MPa for pure gelatin (TG_0) to 948 MPa for the 5% tot’hema concentration (TG_5). As the tot’hema content increases further, Young’s modulus value decreases even more (see Table 1). As demonstrated, the TG_30 film has Young’s modulus of 0.32 MPa (which is very close to 0.35 MPa for PDMS), making it suitable for contact copying. Elastic properties of TG_X films are very important for the production of adaptive micro-optical components.

**Figure 3:**
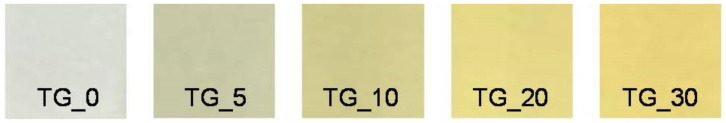
Colors of TG_X films − (gelatin with 0%, 5%, 10%, 20%, and 30% of tot’hema).

The change of color and transparency of the films varied from transparent (pure gelatin) and slightly yellow to yellow with increasing tot’hema content (see Figure 3).

**Figure 4:**
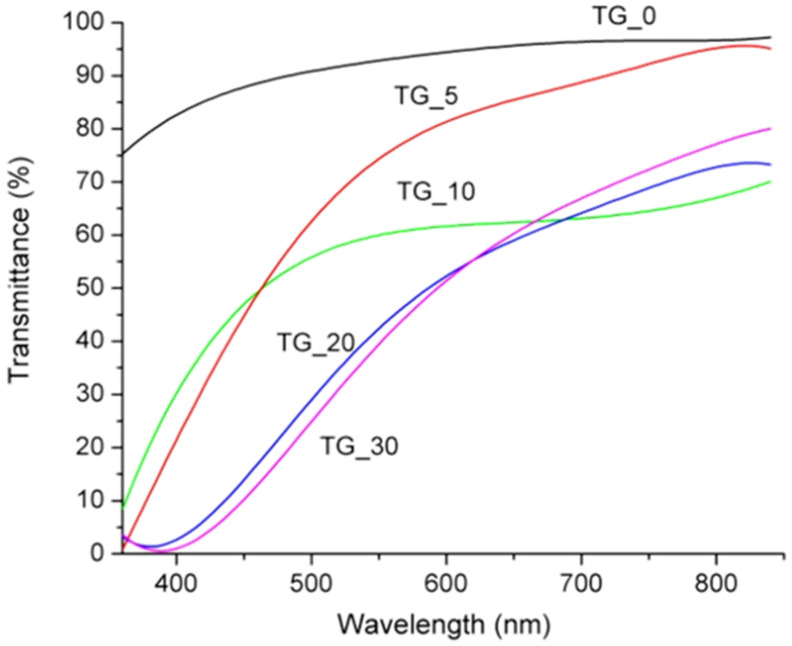
Spectral transmittance of the TG_X films (X− 0, 5, 10, 20, and 30% of tot’hema).

The corresponding spectral light transmittance of the TG_X films as a function of wavelength is presented in Figure 4. Light transmission (in the visible range of 350–800 nm) of the pure gelatin (TG_0) film was above 80%, and the transmission increased with increasing wavelength (see Figure 4). By adding tot’hema, a layer is transformed into a spectral filter whose characteristics strongly depend on the concentration. In all cases, UV radiation is strongly suppressed, making tot’hema-gelatin films an excellent filter for UV-sensitive applications. As we see, pure gelatin film transmits light very well at wavelengths from 350 nm to about 800 nm, but this can be changed at will using appropriate colorants.

**Figure 5:**
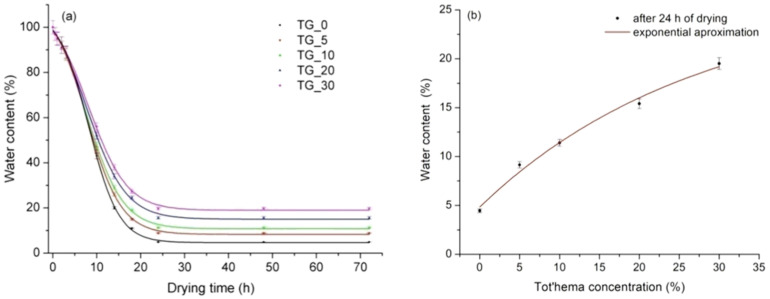
(**a**) Influence of drying time on the water content of TG_X films (X − 0, 5, 10, 20, and 30% of tot’hema); (**b**) The equilibrium water content as a function of tot’hema concentration.

It was noticed that, after the initial rapid decrease of water content for all TG_X films during the drying process (see Figure 5a), the water content remains constant after 24 h of drying. After that, all the other properties remain stable, and the film is ready for further use (e.g., direct laser writing). Pure gelatin film had the lowest water content that exponentially increased with tot’hema content (see Figure 5b). This behavior is consistent irrespective of film thickness. The increase in water content in the presence of a tot’hema can be attributed to the additional hydrogen bonds formed between water molecules and gelatin chains. Water content can influence the physical properties of films (low-humidity films are generally stiff, while films with increased humidity are flexible and malleable). However, relatively high water content does not necessarily mean better physical properties in films [50] but makes the material suitable for a range of applications in biomedicine.

**Figure 6:**
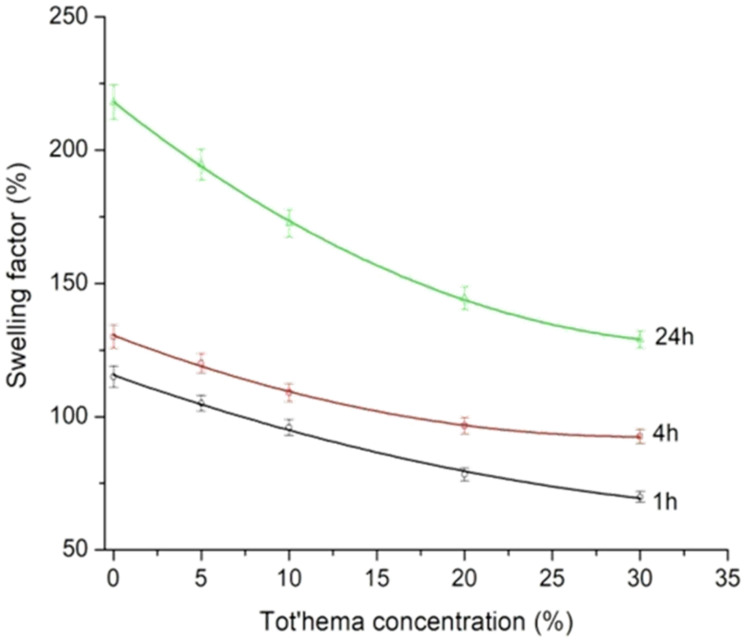
TG_X film (X− 0, 5, 10, 20, and 30% of tot’hema) swelling (during 1, 4 and 24 h) as a function of tot’hema concentration.

The influence of tot’hema concentration on the swelling of TG_X films during 1 h, 4 h and 24 h are shown in Figure 6. The longer the film is immersed in the saline solution, the higher swelling (without deterioration) was observed (after 24 h, the film is fully saturated). Water content and swelling factor of the films were correlated. Opposite to the water content, the film swelling (see Figure 6) decreases with increasing concentration of tot’hema: pure gelatin film has the highest swelling factors (around 220%), while the tot’hema addition reduces the film swelling. This might be due to the limited spaces in film (gelatin network) for absorbing water as already occupied by tot’hema (plasticizer) molecules. The lowest swelling factor for TG_30 is correlated with the largest water amount in the film. Namely, in contact with the film, water diffuses into it, which results in the movement of polymer chains, incorporating between them and the dissolving of added ingredients. In this way, swelling enhances the release effect of some ingredients added to the film, which later diffuse into the system, manifesting different roles (for example, protective) [51].

**Figure 7:**
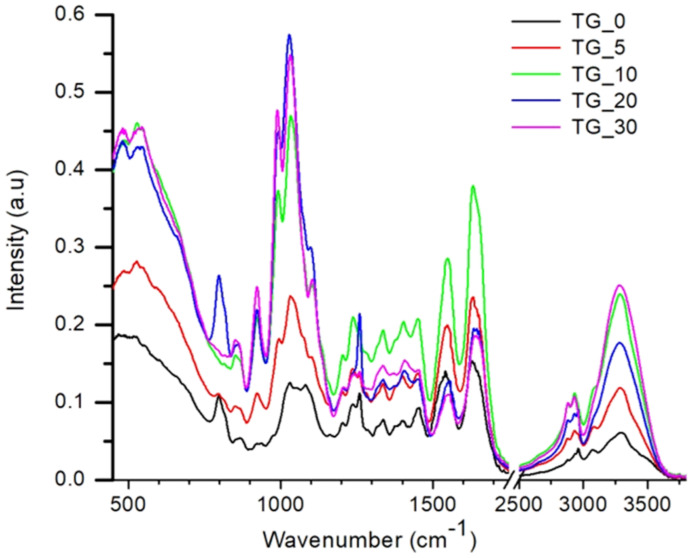
FTIR spectra of TG_X films (X− 0, 5, 10, 20, and 30% of tot’hema).

FTIR analysis was performed to study the interactions between tot’hema and gelatin matrix, i.e., to illustrate possible conformational changes in the gelatin films containing tot’hema. FTIR spectra of TG_X films are presented in Figure 7. A wide band in the range of 3500–3000 cm^−1^ can be attributed to O–H stretching and N–H bending, which are able to form a hydrogen bond with the carbonyl group of the peptide bond in the gelatin, as suggested by a literature review [52,53].

The FTIR spectrum of the pure gelatin film contains characteristic peaks at 3297 cm^−1^ and 2963 cm^−1^, which can be attributed to –OH and –CH vibration stretching peaks. Further, it showed the characteristic amide I, amide II and amide III bands [54]. It is observed that the main absorption of the gelatin film such as C=O stretching at 1629 cm^−1^ (Amide I), N–H bending at 1542 cm^−1^ (Amide II), C–H deformation at 1403 cm^−1^, and C–N stretching (Amide III) at 1237 cm^−1^ remains present in all films.

Compared to pure gelatin film, it was noticed that there are no new peaks in the spectrum of TG_X films. At the same time, the addition of tot’hema caused noticeable changes in the intensity of the Amide I, Amide II, and Amide III band. The peaks characteristic of C–H stretching of CH_2_ and CH_3_ at 2963 cm^−1^ and 2876 cm^−1^ in TG_X films vary significantly in intensity relative to the TG_0. Further, there is a slight shift in the peak’s position, which can be explained by the formation of hydrogen bonds. The shift of these bands to a lower wavelength can be explained by the crosslinking between the components of TG_X films. These data indicate that the polar groups of tot’hema solution interact with the amino acids of the protein chains via inter-and intra-molecular hydrogen bonding and hydrophobic interaction. Therefore, tot’hema adding does not change the chemical composition of gelatin, but it changes the structure of gelatin due to intermolecular hydrogen bonding.

**Figure 8:**
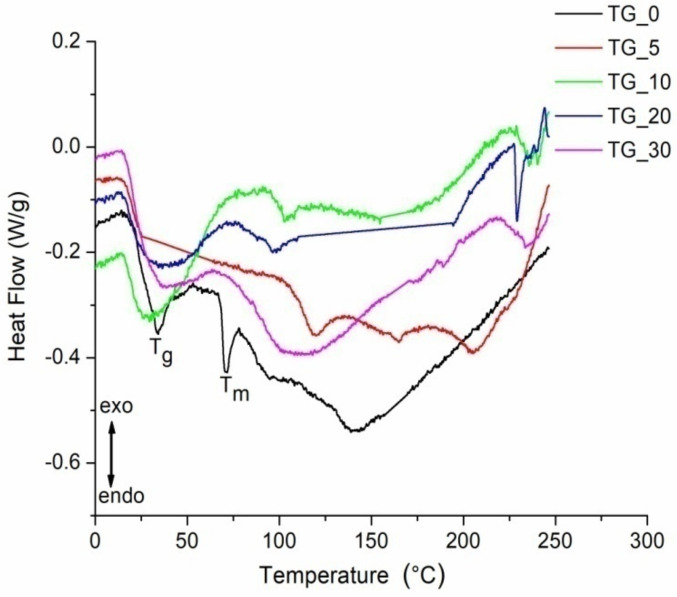
DSC curves of the TG_X films (X− 0, 5, 10, 20, and 30% of tot’hema). Tg and Tm − Endothermic peaks (denoted for pure gelatin film −TG_0) represent glass transition and melting temperature, respectively.

DSC is used to establish thermal stability due to changes in the physical and chemical properties of a material as a function of temperature or time [55]. It is well known that heat can cause structural transitions in protein networks that break chemical bonds [56]. The thermal properties of the film are closely related to their applications. The DSC thermograms of the TG_X films up to a temperature of 250 °C are shown in Figure 8.

The first endothermic peak of pure gelatin film (TG_0) is recorded at a temperature of about 40 °C (see Figure 8). This endothermic peak represents glass transition (T_g_) and is attributed to the transition from the glassy state to rubber. The endothermic peak that follows T_g_, at a temperature around T_m_≈70 °C is attributed to the melting and dissociation of arranged polymer parts. Some authors attribute this peak to the overlap of various processes such as the water evaporation, melting and recrystallization of small and/or imperfect gelatin crystals [57,58].

It was found that the peak temperatures and the enthalpies of these endothermic processes depend on the film preparation and drying conditions [57]. The endothermic peak at about 160 °C represents the gelatin decomposition temperature. It was observed that by adding and increasing the tot’hema content, the endothermic peaks become significantly wider and larger than that of the pure gelatin film. In addition, DSC analysis demonstrated that tot’hema addition, depending on its content, improves gelatin thermal stability. The melting temperature (T_m_) increased when compared to pure gelatin film for all concentrations of tot’hema. T_m_ is typically associated with thermal stability: a high value of T_m_ corresponds to high thermal stability. It is already established that the thermal stability of polymers is related to crosslinking density [59]. In Figure 8, it is noticed that pure gelatin film (TG_0) has the lowest melting temperature (T_m_). This temperature increases after crosslinking gelatin by tot’hema. The highest value of T_m_ was obtained for TG_5 film, while the T_m_ for films prepared with 10–30% of tot’hema show lower values. This behavior is attributed to the hydrophobicity of the gelatin molecular structure resulting from the addition of a large quantity of tot’hema. The improved thermal stability of TG_X films indicates that the temperature range of the use has been extended to about 100 °C, which is very important for practical applications.

To summarize—tot’hema is added to gelatin in order to increase the light absorption, modify its melting temperature and make it permanently elastomeric. Tot’hema concentration was optimized in order to make a compromise between gelatin thermal (e.g., melting temperature), mechanical (flatness) and optical (transparency) properties.

## 4. Discussion

The gelatin-based material described above has a number of useful optical, thermal and mechanical properties, which can be modulated by slight variations of its chemical composition. In this way, it becomes suitable for a range of applications, primarily dealing with micro-mechanics, microfluidics and micro-optics.

The most important property is photoresponsiveness. The photoresponsive gelatin studied here belongs to the class of waste and natural biopolymers (such as alginates and eggshells already reported for the electronics industry [35,36]), which is very important for developing non-toxic, eco-friendly materials. In contrast to the classical photosensitive materials (for example, silver-halide films, photoresists, dichromated gelatin), the response of tot’hema modified material is a consequence of physical, rather than chemical, processes. Here, absorbed light locally heats the material above the melting point, and surface tension pushes the fluidized layer, thus producing a dip (concave, lens-shaped recess), as can be seen in Figure 2. After the light is turned off, rapid cooling “freezes” the surface shape. Due to the low melting point of the gel (approximately 50 °C), the effect is easily achieved by a few milliwatt laser beams and millisecond irradiation times. Depending on the focal point size, micron-sized structures are easily formed. It is important to note that there is no material ablation because the melting point is much higher, and the material is simply displaced [60].

**Figure 9:**
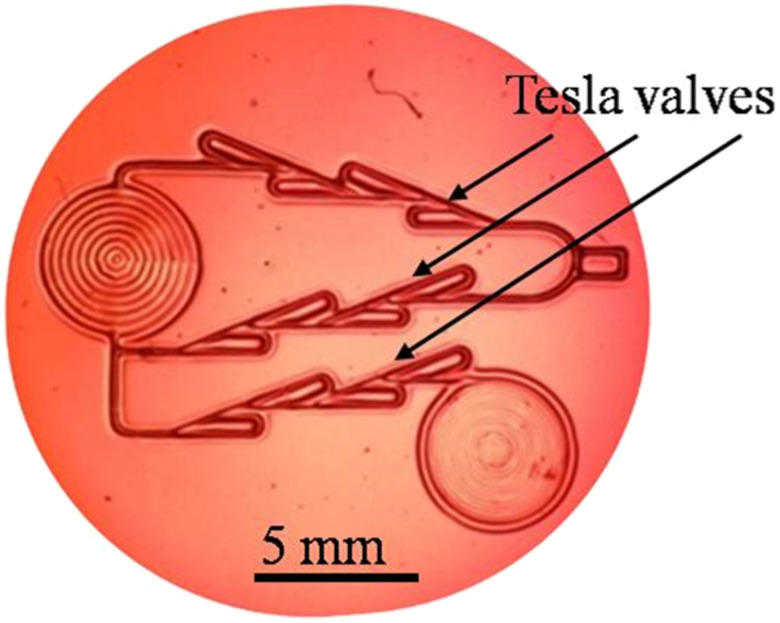
Complex microfluidic channels, with three Tesla valves formed on a tot’hema-gelatin.

This makes the material extremely useful for rapid fabrication of micron-sized features using direct laser writing (DLW)—see Video S1 in a Supplementary Material of this paper demonstrating manufacturing of a complex array of microlenses. By controllably guiding the laser beam across the surface, virtually any shape can be formed (see Figure 9), where a rather complex microchannel structure (with three Tesla valves) is shown [61,62]. The whole pattern was fabricated in less than a minute using a 488 nm laser with 10 mW power (focused by 0.4 NA microscope objective).

Physicochemical processes during interaction with light depend strongly on the laser beam power and focus. The sensitivity threshold is reached at approximately 7.5 nJ/µm^2^ of laser energy, which is easily achieved by focusing the laser beam through the long-working–distance microscope objective (Mitutoyo 20×, 0.4 NA). By appropriately controlling the laser beam size and power, irradiation time and writing speed, features can be fabricated with characteristic widths between 10 µm and 1000 µm and depths up to several hundred micrometers. By using high-power lasers, fabrication time can be significantly reduced to milliseconds for the fabrication of individual microlenses or seconds for more complex structures like the one in Figure 9. Of course, the scanning speed of the laser beam must be increased to prevent material destruction. In most cases, there is no postprocessing, so the optical structures are usable immediately after fabrication.

We have mostly studied optical applications of the material [37,38], which simultaneously offers several key features—optical homogeneity, transparency, surface quality, easy fabrication and environmental friendliness. We have found that tot’hema ingredients mostly determine the material properties. As shown above, spectral absorption and melting point enable tailoring of the material to efficiently absorb the DLW beam. 

The concept of microfabrication using meltable gels can be easily extended by replacing tot’hema with a suitable combination of plasticizers, humectants, preservatives and sensitizing dyes (e.g., betanin, eosin, anthocyanin, etc.) to achieve improved characteristics (faster melting, improved sensitivity, optical transparency after laser processing), which we described elsewhere [37]. In this way, we were able to fabricate a range of optical components, such as diffraction gratings and arrays of positive and negative microlenses.

Gelatin-based materials are biocompatible, and additional chemicals used in this research are non-toxic (even food grade), giving the advantage of quick and natural biodegradability. We have covered a piece of the material with a few centimeters of soil and found that it was completely absorbed, without a trace, within a few days. For some applications, this might be a problem as we observed fungi growing on the material after some time (several days). To prevent that, we added table salt (NaCl), which made it durable and usable over a long period of exposure time to normal room conditions. Normal laboratory conditions (humidity, temperature) do not affect the material properties, and laboratory illumination does not diminish the material sensitivity (i.e., the material does not have to be kept in darkness). Dust is the major problem because of the stickiness of the film, and the material surface must be protected by an additional cover glass.

Softness is yet another possible disadvantage. Surfaces should not be scratched or punched with hard objects as this will certainly damage the structure. On the positive side, films can be easily cut, peeled from the substrate, stacked together and transferred. Mechanical hardness can be further significantly increased by removing the plasticizer (by submerging the films in cold water and letting the plasticizer diffuse out) or by hardening (tanning) with, e.g., alum [63]. In addition, we have been able to use tot’hema-gelatin as a template for contact copying into harder materials (epoxies, photopolymer composites). 

The usable temperature range is limited by the melting point to somewhere between 50 °C and 100 °C, depending on tot’hema concentration. For the majority of applications, this is quite satisfactory and makes the material usable in the widest range of applications: lab on a chip, sensors and micro-optics. We have also found that above the critical power density of the laser beam, material locally carbonizes and can be used as a blocking filter [39] and, possibly, as an electrical conductor.

Finally, the fabrication cost is really low. Materials used here are cheap and ubiquitous. Most of them can be found in the kitchen, while the fabrication of layers is straightforward and simple. Production methods are well known in the film industry and can be used both in low- and large-scale production.

## 5. Conclusions

To conclude, here we disclosed an innovative combination of well-known substances producing really versatile material for safe, real-time, low-cost, rapid microfabrication. Physicochemical properties of the material have been thoroughly studied, aiming to establish operational limits and optimize properties for the desired application. Application potential is high due to the simplicity of fabrication of highly complex structures, good optical properties, and manufacturability by direct laser writing without any further postprocessing, micrometers resolution, elastomeric characteristics, environmental friendliness, and non-toxic character—a rare combination of properties concentrated in a single material. Elasticity, uniform and homogeneous surface and optical transparency were achieved, making the material suitable for the production of adaptive micro-optical components and protective filters. The temperature range of the material’s applicability is extended up to 100 °C.

## Figures and Tables

**Table 1:** The film thickness, stress at break, strain at break and Young’s modulus.

Film	Tot’hema (%)	Thickness (µm)	Stress at Break (MPa)	Strain at Break (%)	Young’s Modulus (MPa)
TG_0	0	25 ± 10	58 ± 1	2 ± 0.1	1933 ± 40
TG_5	5	35 ± 10	37.92 ± 0.76	4 ± 0.2	948 ± 10
TG_10	10	50 ± 10	4.89 ± 0.10	40 ± 2	12.23 ± 0.06
TG_20	20	100 ± 10	1.71 ± 0.03	140 ± 6	1.22 ± 0.02
TG_30	30	200 ± 10	0.72 ± 0.01	224 ± 10	0.32 ± 0.01

Results are expressed as mean value ± standard deviation.

## Data Availability

Not applicable.

## Supplementary Materials

The following supporting information can be downloaded at: https://www.mdpi.com/article/10.3390/polym14122350/s1, Video S1: Microoptics fabrication.
